# The Pathology of General Paralysis of the Insane

**Published:** 1906-02-10

**Authors:** 


### HOSPITAL CLINICS.

### THE PATHOLOGY OF GENERAL PARALYSIS OF THE INSANE.

THE Morison lectures recently delivered before the Royal College of Physicians, Edinburgh, were devoted to the Pathology of General Paralysis of the Insane, the lecturer being W. Ford Robertson, M.D., director of the Laboratory of the Scottish Asylums. The lecturer began by referring to the prevalence and recent increase of general paralysis in this and other countries, which caused the deaths of nearly 2,000 persons annually in English and Scottish asylums alone. In spite of its well-defined clinical course and morbid anatomy, there was no satisfactory explanation of its cause. Its alleged relation with syphilis presented many difficulties, and there might be traced in the recent literature of the subject a growing scepticism as to its being a syphilitic or even a para-syphilitic manifestation. The lecturer then described the morbid changes in the brain which, with the clinical observations made by Dr. L. C. Bruce, of Murthly Asylum, led him six years ago to the opinion that general paralysis depended on an active bacterial toxæmia, the invasion probably starting from the alimentary canal. In 1902, along with Drs. McRae and Jeffrey, he commenced a bacteriological investigation and discovered that an organism having a close resemblance to the Klebs-Löffler bacillus was constantly present and very prominent in the alimentary and respiratory tracts of persons dying from general paralysis. The morphology and staining reactions of this organism were similar to those of the bacillus of diphtheria, and when stained by Neisser's method it showed meta-chromatic granules. Inoculation experiments were unsuccessful, but rats fed on cultures of the bacillus had died after exhibiting tremor and paralytic symptoms; while a goat infected by Dr. Bruce had suffered from symptoms resembling a congestive attack. In the tissues of these animals the invasion of the bacilli could be seen exactly as in the human subject, and vascular changes comparable to those met with in general paralysis were produced. These diphtheroid bacilli could be demonstrated penetrating the mucous membrane of the bronchial and intestinal walls, and it had been possible to cultivate them from the brain in 9 cases of general paralysis out of 23 examined. They had since been shown to be present in the walls of the cerebral vessels and in the blood, cerebro-spinal fluid and urine of the living general paralytic, especially during congestive attacks. In these cases the bacilli were more or less altered in form and staining reactions indicating that when these organisms gained access to the blood they were rapidly destroyed by phagocytic and lysogenic action. In order to test this, pure cultures of bacilli from cases of general paralysis were subjected to the action of living blood from general paralytics and also from healthy individuals or patients suffering from other conditions. It was found that the bacilli were rapidly altered by contact with living blood both by the leucocytes and by the plasma, but chiefly by the former, and that the blood of the general paralytic is much more active in this respect than that of a healthy individual. After an incubation of three hours the leucocytes of the general paralytic took up a much larger proportion of the organisms than in control experiments, which might prove of some value as a diagnostic reaction, though it was probable that a direct bacteriological examination was more reliable. In a doubtful case if one succeeded in obtaining a pure culture of the organism from the blood or cerebro-spinal fluid or in demonstrating the bacillus in preparations from these, the disease was general paralysis.

These observations on the alteration of the bacilli by the blood had led to the recognition of large numbers of altered organisms in the tissues of persons dying from general paralysis and also those found in the blood and cerebro-spinal fluid during life. It had also indicated the method of culture from the living fluids which had at first been unsuccessful. This was due to the bactericidal power of the leucocytes and was overcome by allowing the inoculated tubes to stand at the normal temperature for some time before putting them in the incubator, thus getting rid of the cells chiefly concerned. In this way it had been possible to obtain pure cultures of the diphtheroid bacillus from the blood in four cases and from the cerebro-spinal fluid in two others. The organism, which it was proposed to name Bacillus paralyticans, was extremely polymorphic, and when seen in cases of general paralysis during a congestive attack frequently assumed a thread form which could readily be produced in cultures by growing them at a slightly higher temperature. The meta-chromatic granules were rarely seen in the bacillus as isolated from general paralytics, but were present in organisms obtained from cases of tabes dorsalis, and made their appearance when the growths were kept at a low temperature. While the relations of B. paralyticans to the Klebs-Löffler bacillus of diphtheria were not settled, Dr. Robertson expressed the opinion that the organisms were not identical, though the former might be a form of the latter of much lower virulence.

In considering the etiology and pathogenesis of general paralysis and tabes dorsalis in the light of the above facts, the lecturer expressed the opinion that the essential cause of these conditions was the invasion of the perivascular lymphatics of the brain and spinal cord by B. paralyticans. Syphilis must be regarded as an active predisposing cause, but there were facts to strongly contra-indicate its true causal relation to these conditions. In a certain proportion of cases capable observers failed to obtain any evidence of syphilitic infection, while cases where tertiary and also secondary syphilitic lesions had coincided with general paralysis had been recorded, the former being at once benefited by treatment, while the latter had followed its usual course. Patients suffering from general paralysis had even been known to contract syphilis. Syphilis was, however, an important agent in lowering the power of defence against bacterial invasion, as were also chronic alcoholism and the excessive use of nitrogenous foods. It was only in persons whose mucous membranes had been the seat of a long-standing inflammatory process that the bacillus could begin to invade the tissues. Until such invasion took place there was probably but little toxic action. The successive waves of invasion were met by the leucocytes and tissue-cells but gradually attained the perivascular lymphatics of the central nervous system, setting up the changes of the vessels and degeneration of the pyramidal cells of the cerebral cortex. In tabes it had been shown that the nervous elements were protected by their neurilemma until their point of entrance into the spinal cord by the posterior nerve roots. The invasion in this case was probably from the bladder, in which the bacillus was often present in a state of pure culture. The chief change was the well-known degeneration of the posterior columns; but vascular and cellular changes comparable to those of general paralysis were also met with in other parts of the cord. That Bacillus paralyticans was not a local organism was proved by its occurrence in material derived from various asylums in this country and abroad.

In combating these diseases something might be done, Dr. Robertson thought, in preserving susceptible persons from the action of the organism. There was also a hope that an anti-serum might be obtained which would prove of value in treatment. Treatment on these lines was about to be instituted at Morningside Asylum, and though the results of such treatment in other diseases had with a few exceptions been disappointing, it was intended to give it a trial.

